# Identification of tubulin gene variants in patients with dandy-walker malformation: expanding the spectrum of tubulinopathies

**DOI:** 10.1186/s13039-026-00772-5

**Published:** 2026-06-17

**Authors:** Katsuya Ueno, Koichiro Higasa, Mikio Hayashi, Haruna Isozaki, Mayuko Miyata, Nobuaki Naito, Yi Li, Junichi Takeda, Masahiro Nonaka

**Affiliations:** 1 Department of Neurosurgery, Kansai Medical University, 2-5-1 Shinmachi, Osaka 573-1010 Hirakata, Japan; 2 Department of Genome Analysis, Institute of Biomedical Science, Kansai Medical University, Hirakata, Osaka Japan; 3 Department of Physiology, Kansai Medical University, Hirakata, Osaka Japan

**Keywords:** Tubulinopathy, Dandy-Walker malformation, Cerebellar hypoplasia

## Abstract

Dandy-Walker malformation (DWM) is a condition characterized by a cyst in the posterior cranial fossa contiguous with the fourth ventricle, combined with complete or partial agenesis of the cerebellar vermis; and elevation of the cerebellar tentorium, torcular Herophili, and transverse sinuses. DWM has been linked to specific genetic variants, with pathogenic or likely pathogenic variants reported in *FOXC1*, *ZIC1*, and *ZIC4*. Variants in the *TUBB2B* and *TUBB3* genes are associated with abnormalities in tubulin, leading to cerebellar hypoplasia. In our study, three patients diagnosed with DWM underwent whole-genome analysis using next-generation sequencing, and two were found to have heterozygous variants in the tubulinopathy-associated genes *TUBB2B* and *TUBB3*, respectively. These findings indicate that some cases previously diagnosed as DWM may fall under the spectrum of tubulinopathies associated with cerebellar hypoplasia.

**Supplementary Information:**

The online version contains supplementary material available at https://doi.org/10.1186/s13039-026-00772-5.

## Introduction

Dandy-Walker malformation (DWM) is a congenital brain malformation characterized by a cyst in the posterior fossa contiguous with the fourth ventricle, in conjunction with partial or complete agenesis of the cerebellar vermis; along with elevation of the cerebellar tentorium, transverse sinus, and torcular Herophili [1–3]. The prevailing hypothesis regarding the cause of DWM suggests that abnormal development in the fetal rhombencephalic roof plate leads to posterior projection and cystic enlargement of the fourth ventricle, triggering dysplasia of the cerebellar vermis. DWM is also characterized by other abnormalities and malformations of the central nervous system, including agenesis of the corpus callosum, occipital encephalocele, polymicrogyria, and heterotopia, which present in 30–50% of individuals [4]. Genetic factors, including chromosomal abnormalities, have been implicated in its pathogenesis, with an estimated 16.3% of isolated DWM cases involving chromosomal deletions [5]. Of these, PHACE syndrome, trisomy 18, and Joubert syndrome are among the more common genetic syndromes [6].

Previous studies have identified several genes involved in DWM, including *ZIC1*, *ZIC4* [7–9], , and *FOXC1* [10], although only a small number of cases have been attributed to mutations involving these genes. Grinberg et al., initially identified a critical locus for DWM associated with *ZIC1* and *ZIC4* heterozygous deletions in individuals with a 3q24 deletion [11]. Studies using zebrafish models further demonstrated that the absence of *Zic* function resulted in a reduction in the proliferation of rhombencephalic progenitor cells, thereby impairing dorsal roof plate development in the fourth ventricle [12]. The *FOXC1* gene, located on chromosome 6p25.3, also plays a critical role in normal cerebellar development, and has been implicated in DWM, as well as other posterior fossa malformations [10].

Whole-genome sequencing (WGS) has arisen as a valuable tool exploring genetic abnormalities in DWM, particularly as the costs and time required for sequencing have significantly decreased in recent years. This technique holds significant potential for uncovering new genetic factors in cases of DWM where the etiology remains unknown.

## Methods

### Editorial policies and ethical considerations

The study protocol was approved by the Ethics Committee of the Kansai Medical University (No. 2020330). Written informed consent was obtained from all participants or participants’ parents.

### Patients

DWM is classically defined by the presence of morphologic features of posterior fossa enlargement (venous sinus commissure and elevation of the cerebellar tent), with varying degrees of hypoplasia of the cerebellar vermis, as well as cyst-like enlargement of the fourth ventricle. When imaging findings are mild or atypical, it is sometimes called Dandy-Walker variant. In addition to the classical definition, Whitehead et al., proposed that a large tegmentovermian angle, obtuse fastigial recess, and inferolateral displacement of the taenia-tela choroidea and choroid plexus as criteria for the diagnosis of DWM [3]. In addition to the traditional diagnostic criteria proposed by Klein et al., we also incorporated the quantitative MRI-based criteria proposed by Whitehead et al., to identify patients with DWM at our institution [2, 3]. All of these patients underwent whole genome analysis using a next-generation sequencer after obtaining written consent for DNA analysis from the patients, followed by blood sampling. After extracting genomic DNA from the patients’ peripheral blood cells, WGS was conducted using the Illumina NovaSeq 6000 sequencer (Illumina Inc., San Diego, CA, USA). The sequence reads were aligned to the reference genome (GRCh37/hg19) using Burrows-Wheeler Aligner [13]. Downstream analyses, including marking duplicates, base quality recalibration, haplotype call, joint base calling, and variant quality score recalibration, were processed using Picard and GATK version 3.8 according to the GATK Best Practice recommendations [14]. Pathogenic variants were determined by referring to public databases, such as the Human Genetic Variation Database (HGVD) [15], jMorp [16], Clinical Variation (ClinVar), and dbSNP. The identified variants were subjected to confirmatory testing using Sanger sequencing.

## Results

We performed WGS on three patients with DWM and two relatives using next-generation sequencing. Among the three patients, one (ID: BM10) carried a pathogenic *TUBB3* mutation, another (ID: BM74) had a pathogenic variant in *TUBB2B*, and the remaining patient (ID: BM65) exhibited a partial deletion of the long arm of chromosome 12. The two relatives showed no abnormalities; one (ID: BM09) was the mother of the patient with the *TUBB3* mutation, and the other (ID: BM64) was the mother of the case with the chromosome 12 deletion. We have provided the essential sequencing quality metrics (Supplementary Tables 1 and 2).

## Case 1

**Fig. 1:**
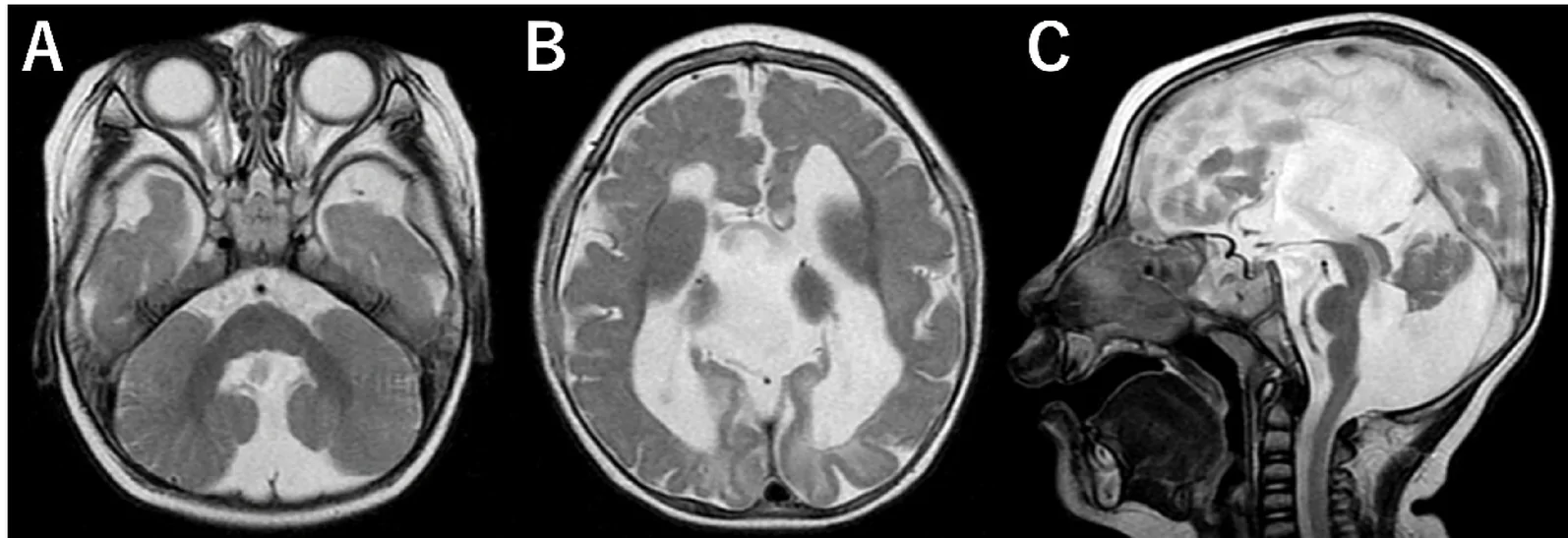
A, B: T2-weighted axial images, and C: T2-weighted sagittal images taken at age 8 months, showing cerebellar vermis hypoplasia (A, arrow), basal ganglia malformation (B, arrow), and corpus callosum dysplasia (C, arrow). The cerebellar tent is deviated upward and the posterior fossa is enlarged (C, arrowhead)

**Fig. 2:**
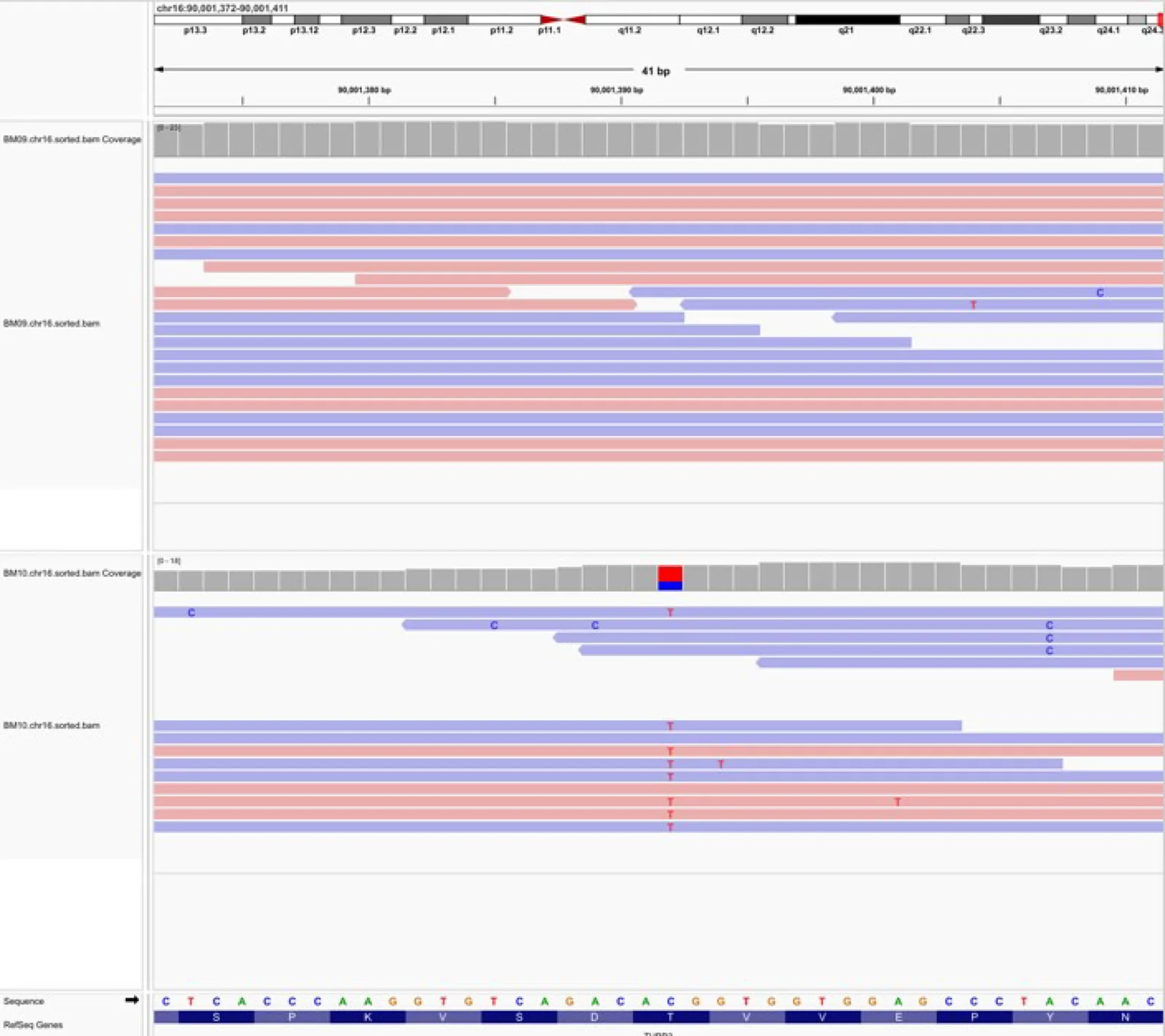
IGV visualization of the *TUBB3* c.317 C > T (p.T106M) variant Integrative Genomics Viewer (IGV) snapshot showing aligned whole-genome sequencing reads at chr16: 90,001,392 (GRCh37/hg19). The upper panel represents BM09, in which only the reference allele (C) is detected. The lower panel represents BM10, demonstrating a heterozygous substitution with both reference (C, blue) and alternate (T, red) reads. Read depth confirms sufficient coverage without strand bias. This variant corresponds to a nonsynonymous SNV in TUBB3, resulting in the amino acid substitution p.T106M.

Case 1 (ID: BM10) was a 2-year-old boy born by normal delivery with enlarged ventricles noted prenatally on ultrasonography and magnetic resonance imaging (MRI). He was unable to hold his head up by age 2 years, and had severe developmental delays. While the patient fulfilled MRI findings suggestive of Dandy–Walker variant—including cerebellar vermis hypoplasia, fourth ventricle enlargement, and upward deviation of the cerebellar tent—several additional abnormalities demonstrated on MRI, such as basal ganglia malformation and corpus callosum dysplasia, are also consistent with the broader spectrum of tubulinopathy-associated malformations (Fig. 1). The patient had not undergone surgical treatment and had been treated with an anti-epileptic drug and adrenocorticotropic hormone therapy for West syndrome. Whole-genome analysis revealed a heterozygous TUBB3 variant: NM_006086.4:exon4:c.533 C > T (p.Thr178Met), which is classified as pathogenic in ClinVar. This variant is listed under ClinVar record RCV000254864.2, with a submission by GeneDx (SCV000322117.6) describing it as a de novo occurrence in an individual with brain malformations (Fig. 2). The TUBB3 p.T178M variant has conflicting interpretations of pathogenicity in ClinVar, with submissions classifying it as either “Pathogenic” or “Likely pathogenic.” According to the guidelines from the American College of Medical Genetics and Genomics and the Association for Molecular Pathology (ACMG/AMP), this variant meets the criteria for classification based on the evidence codes PS1, PM1, PM2, PP2, and PP3. The mother (ID: BM09) tested negative for the variant, whereas the father has not undergone genetic testing. Therefore, a de novo origin cannot be definitively confirmed; however, given the mother’s normal genotype and phenotype, the TUBB3 variant in the child is most likely de novo. The Sanger sequencing results for Case 1 (BM09 and BM10) are shown (Fig. S1), and their corresponding WGS-based genomic views are presented.

## Case 2

**Fig. 3:**
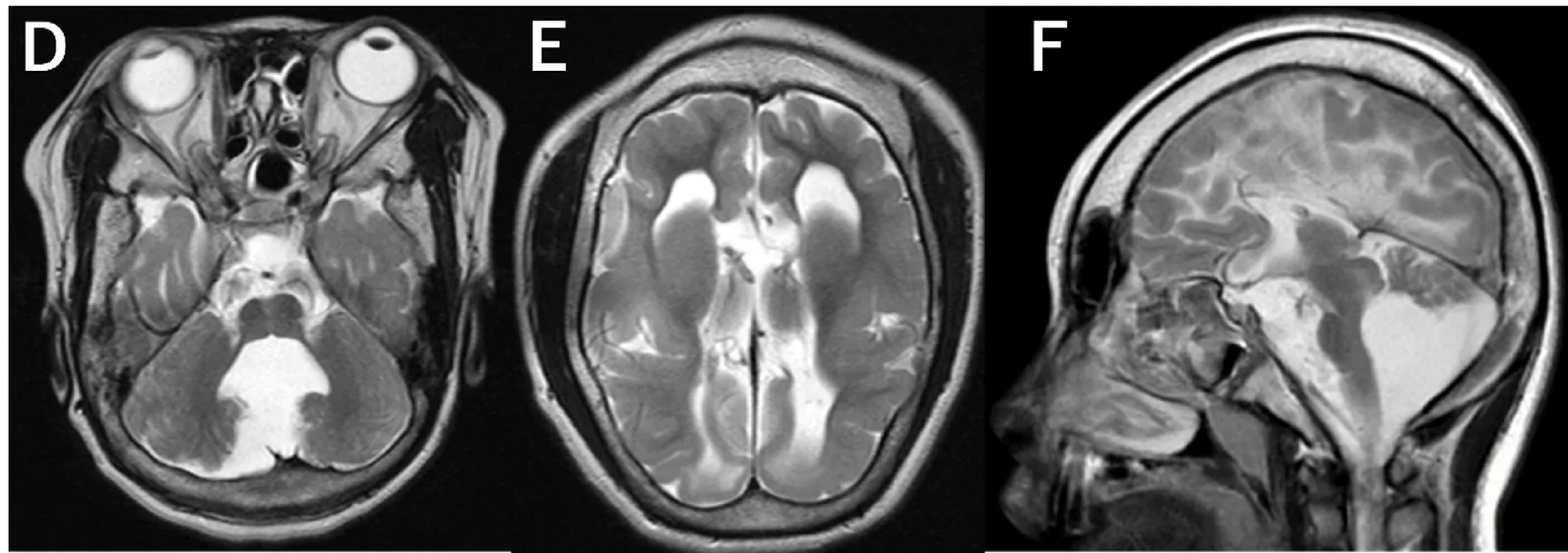
Brain MRI findings of Case 2 with Dandy–Walker malformation. D, E: T2-weighted axial images, and F: T2-weighted sagittal images at age 13. These images show complete cerebellar vermis defect (D arrow), basal ganglia malformation (E arrow), corpus callosum dysplasia (F arrow), and a giant cyst contiguous to the fourth ventricle (F white arrowhead)

**Fig. 4:**
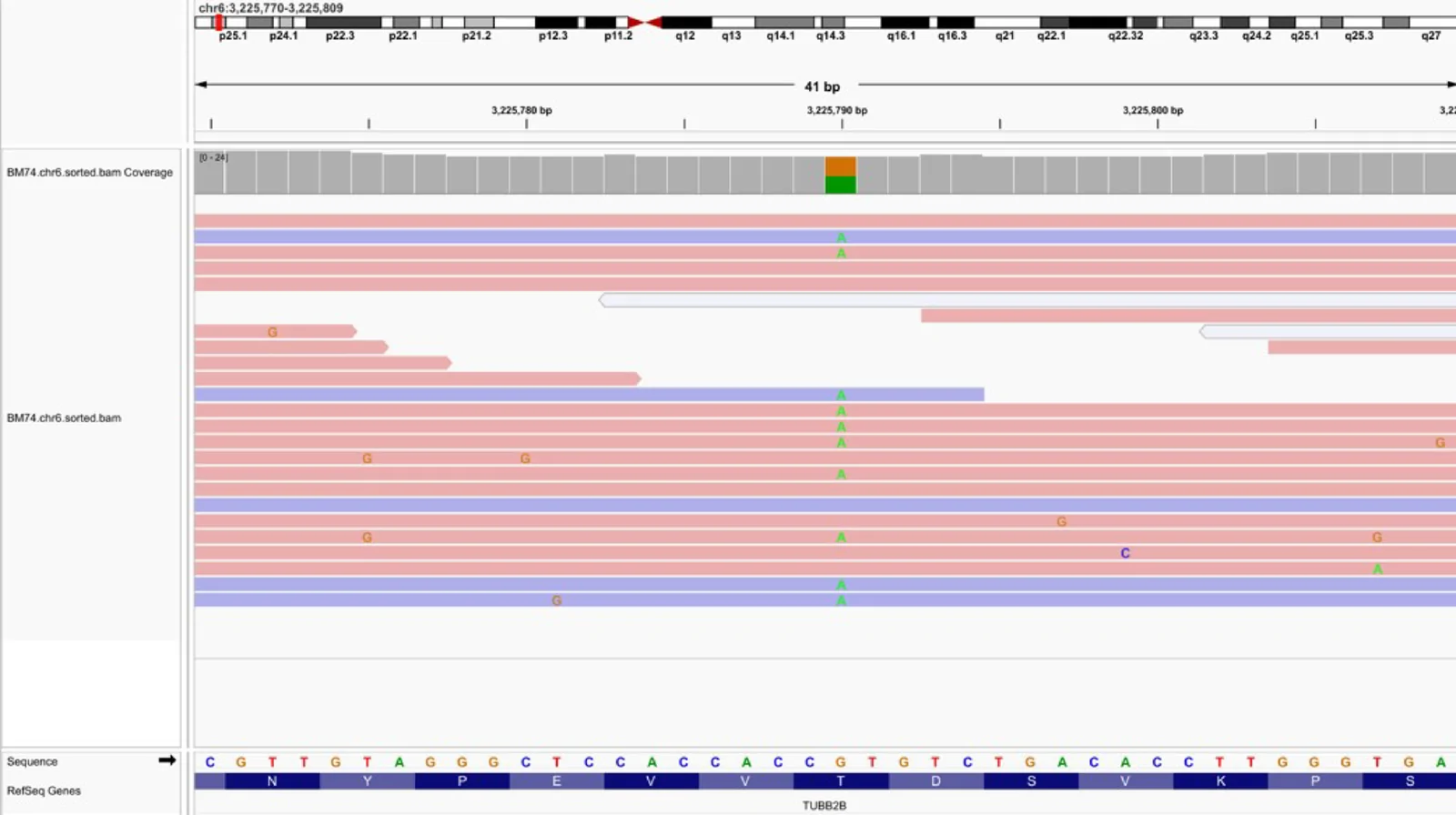
IGV visualization of the *TUBB2B* c.533 C > T (p.Thr178Met) variant. Integrative Genomics Viewer (IGV) snapshot displaying aligned whole-genome sequencing reads at chr6: 3,225,770–3,225,809 (GRCh37/hg19), corresponding to exon 4 of TUBB2B (NM_178012.4). BM74 shows a heterozygous C>T substitution at chr6: 3,225,790, supported by multiple reads with sufficient coverage. The nucleotide change produces a missense mutation in TUBB2B, leading to an amino acid substitution p.Thr178Met.

Case 2 (ID: BM74) was a 15-year-old girl who had undergone implantation of a ventriculoperitoneal shunt in another hospital at 6 months of age. She was severely developmentally delayed and suffered from intractable epileptic seizures. MRI revealed cerebellar hypoplasia and corpus callosum dysplasia (Fig. 3), similar to the findings observed in Case 1. Whole-genome analysis identified a TUBB2B variant, NM_178012.5:c.533 C > T (p.Thr178Met), which is classified as pathogenic in ClinVar (RCV ID not specified) (Fig. 4). This variant is associated with autosomal dominant inheritance. The zygosity was heterozygous. The TUBB2B p.Thr178Met (c.533 C > T) variant has been classified as “Pathogenic” by GeneDx and as “Likely pathogenic” by the Genetic Services Laboratory at the University of Chicago, based on clinical testing and in accordance with ACMG guidelines. Her parents were not tested for the variant. In Case 2, parental DNA was unavailable; therefore, we were unable to determine whether the variant had arisen de novo. This remains a limitation of the present study. We validated this variant by Sanger sequencing (Fig. S2), and we also provided the corresponding WGS-based genomic view.

## Discussion

Since the discovery of *TUBA1A* as the causative gene for malformations of cortical development in 2007, the recognition of tubulinopathies has expanded, establishing mutations in α-tubulin and β-tubulin subunits as the primary causes of various cortical dysplasias [17]. These mutations impact the microtubule dynamics crucial for neurodevelopmental processes, such as neuronal migration, axon and dendrite growth, and synaptic connections between Purkinje cells [18]. Studies have reported a high prevalence of corpus callosum aplasia and cerebellar dysplasia in tubulinopathy cases, with genetic mutations spanning multiple tubulin isoforms, including α-tubulin (*TUBA1A*), β-tubulin (*TUBB2A*, *TUBB2B*, *TUBB3*, and *TUBB4A*), and γ-tubulin (*TUBG1*) [17, 19].

A previous report described a patient with DWM harboring a *TUBA1A* variant [20]. In the present study, two patients with DWM were identified as having *TUBB2B* or *TUBB3* variants, further supporting the association between DWM and tubulinopathies. Among these, the *TUBB3* T178M [533 C > T] variant, a mutation previously documented in five cases with reported cerebellar hypoplasia [21], was detected in one patient. Functional studies have shown that *TUBB3* T178M variants affect tubulin stability, potentially contributing to microtubule dysfunction and subsequent cerebellar hypoplasia [22]. Although the *TUBB3* R262C-substituted mouse model did not trigger any phenotypic abnormalities in heterozygotes, the lethal effects in homozygotes highlight the functional significance of tubulin stability in neurodevelopment [23].

The findings of this study suggest that DWM cases with partial or complete agenesis of the cerebellar vermis may be associated with tubulin gene abnormalities, specifically involving the *TUBB2B* and *TUBB3* genes. Although cerebellar hypoplasia, enlargement of the fourth ventricle, and elevation of the tentorium cerebelli were present in our cases, the overall neuroimaging pattern did not fully match the classic triad of DWM. Instead, the findings showed overlap with the broader spectrum of tubulinopathy-associated malformations, which are known to exhibit considerable anatomical variability. These cases could represent an intersection between DWM and tubulinopathies, indicating a broader phenotypic spectrum of tubulin-related neurodevelopmental disorders. Future studies with *TUBB3* T178M knock-in mouse models could provide insights into the phenotypic consequences of tubulin dysfunction in cerebellar development, potentially expanding our understanding of the genetic etiology of DWM.

## Conclusion

In this study, whole-genome analysis performed on two patients with DWM using next-generation sequencing revealed genetic mutations in *TUBB2B* and *TUBB3*, which are known to be involved in tubulinopathies. The further identification and characterization of tubulin gene mutations in patients with DWM may lead to an expanded spectrum of tubulinopathies.

## Supplementary Information


Supplementary Material 1



Supplementary Material 2


## Data Availability

No datasets were generated or analysed during the current study.
