# Supplementary material for: Identification of tubulin gene variants in patients with dandy-walker malformation: expanding the spectrum of tubulinopathies
Source: Mol Cytogenet. 2026 Jun 17;19:30. doi: 10.1186/s13039-026-00772-5 (PMC13528077; doi:10.1186/s13039-026-00772-5)
Supplement: Supplementary file 1 [file 13039_2026_772_MOESM2_ESM.docx]

**Supplementary Figure Legends**

Figure S1. Sanger sequencing chromatograms of BM09 (wild-type control) and BM10 confirming a heterozygous substitution.

(A) Chromatogram from BM09 showing a single reference T peak at the target position (black arrow), indicating a wild-type sequence without nucleotide substitution. (B) Chromatogram from BM10 showing overlapping C and T peaks at the same genomic position (black arrow), consistent with a heterozygous nucleotide substitution. This confirms the presence of the variant detected by whole-genome sequencing.

Figure S2. Sanger sequencing chromatogram of BM74 confirming a heterozygous substitution.

The chromatogram shows a double peak consisting of overlapping C and T signals at the target genomic position (black arrow), indicating the presence of both reference and alternate alleles. This pattern is consistent with a heterozygous nucleotide substitution.

**Supplementary Table Legends**

Supplementary Table 1. Mapping statistics of whole-genome sequencing for the five samples.

Mapping statistics were calculated based on alignment to the GRCh37/hg19 reference genome.

Coverage indicates the average sequencing depth; >10× and >20× indicate the proportion of genomic bases covered by at least 10 and 20 reads, respectively.

Supplementary Table 2. Mutation details identified by whole-genome sequencing.

Genomic positions are based on the GRCh37/hg19 reference genome. For each variant, chromosome, position, reference/alternate allele, Phred-scaled quality score, and sample-specific genotype information are shown. Genotype fields follow the VCF format: GT (genotype), AD (allelic depth), DP (read depth), GQ (genotype quality), PL (Phred-scaled likelihoods), and SAC (strand-specific allele counts). Functional annotation includes gene symbol, predicted exonic function, amino acid change (AAChange.refGene), dbSNP ID (rs ID), and population allele frequencies (jMorp and HGVD).

Supplementary Table 3. List of deleted genes.

Summary of genes located within the deleted chromosomal region 12q23.3–q24.11, including gene symbols, mode of inheritance (MOI), OMIM identifiers, and associated phenotypes (PHE) recorded in the OMIM database. “NA” indicates genes with no reported inheritance pattern or phenotype description currently available in OMIM. Both autosomal dominant (D) and autosomal recessive (R) inheritance patterns are shown when applicable.
